# Time-Space Simulation, Health Risk Warning and Policy Recommendations of Environmental Capacity for Heavy Metals in the Pearl River Basin, China

**DOI:** 10.3390/ijerph19084694

**Published:** 2022-04-13

**Authors:** Feng Liang, Yujie Pan, Hongxia Peng, Min Zeng, Changsheng Huang

**Affiliations:** 1 Department of Primary Education, Normal College of Jishou University, Jishou 416000, China; liangfeng@jsu.edu.cn; 2 College of Environmental Sciences and Engineering, Peking University, Beijing 100871, China; 2101112207@stu.pku.edu.cn; 3 Department of Geography, School of Geography and Information Engineering, China University of Geosciences, Wuhan 430074, China; penghx@cug.edu.cn; 4 Wuhan Geological Survey Center of China Geological Survey, Wuhan 430205, China; huangzhangsheng@mail.cgs.gov.cn

**Keywords:** heavy metals, environmental capacity, health risk warning, Pearl River Basin

## Abstract

In China, the environmental capacity problem of heavy metals has long been hidden in the Pearl River Basin creating a contradiction between the economic development and environmental health. Thus, this research calculated the environmental capacity of heavy metals in the agricultural land of the urban agglomeration in the Pearl River Basin, evaluated the health risk warning capacity using a comprehensive index. The results showed that the static capacity order of heavy metals in the study area was As > Pb > Zn > Cr > Hg > Cu > Ni > Cd. The dynamic capacity showed an upward trend, and it fluctuated in some cities. The remaining capacity of Cr and Ni was relatively poor, and the comprehensive soil quality index of the Pearl River Basin was 0.64. The pollution level was of grade IV, which belongs to the medium capacity, but the soil pollution risk still existed, which threaten the health of local resident. In this regard, this study also put forward some countermeasures for pollution control. Thus, studying the soil heavy metal environmental capacity can provide a reference for heavy metal pollution control and health risk early warning in the Pearl River Basin.

## 1. Introduction

As the link between the water and soil circle layers, the river basin not only plays an important role in protecting the environment and maintaining ecological balance, but it is also the basis for ensuring good human health [1]. However, heavy metal pollution has become the main pollutant in river basins and threatens human health and ecosystems [2]. Heavy metals can enter the human body through ingestion, dermal contact, or breathing, and they can threaten health. Low concentrations of these elements can cause various health issues, such as renal dysfunction, endocrine disorders, reproductive dysfunctions, and cancers. However, with the rapid development of industrialization and urbanization, humans ignore the risk that the heavy metal pollutants, which are emitted by social and economic activities, pose to the environmental soil capacity, which leads to a conflict and imbalance between economic development and environmental health [3].

The soil environmental capacity refers to a certain environmental unit that follows the environmental quality standards in a certain period to ensure the output and quality of agricultural products without causing environmental pollution that exceeds the maximum load the soil can take [4,5]. Once this load is exceeded, the soil faces heavy metal pollution, which leads to a variety of environmental problems and threatens human health [6,7]. It can be seen that the study of the soil heavy metal environmental capacity is important for understanding soil heavy metal pollution, health risk warning and the subsequent pollution prevention and remediation, and it is also a positive response to the integration of the scientific research resources in the soil pollution Prevention Action Plan issued by the State Council of China in 2016 [8].

In recent years, scholars have continuously carried out studies on the soil environmental capacity [9,10,11]. The early research mainly focused on the construction of a soil heavy metal environmental capacity model. For example, Zhang et al. took the soil ecosystem as a main research center to obtain the risk screening values of Hg, As, Cr, Pb, and Cd in a meadow brown soil in the study area, and they also carried out a field material balance experiment. Based on a soil capacity mathematical model, the soil dynamic capacity was predicted [12]. Xia et al. used multiple indicators to determine the critical content of soil heavy metals, obtained the soil dynamic capacity, and established a corresponding dynamic environmental capacity model, but this model did not involve the study of the soil heavy metal environmental capacity of the agricultural land in urban agglomerations [13]. Driven by the agglomeration effect, all kinds of large-, small-, and medium-sized enterprises keep flocking to urban agglomeration areas, which not only make an important contribution to the economic development of the urban agglomeration, but they also lead to serious environmental pollution problems [14,15]. With the accelerating industrialization and urbanization process, the problem of the soil heavy metal environmental capacity of the agricultural land in urban agglomerations has become critical [16]. Based on incomplete statistics, the discharge of industrial wastewater, industrial waste gas, and industrial solid waste in China’s urban agglomerations accounts for more than 67% of the total discharge amount in the country, respectively, and it is concentrated in eastern coastal urban agglomerations [17]. Based on these findings, researchers estimated, predicted, and evaluated the environmental capacity of heavy metals in individual urban soils in various urban agglomerations [18]. For example, Fu et al. estimated the environmental capacity of heavy metals in the Lishui area and analyzed the factors affecting the environmental capacity distribution [19]. Chen et al. used the heavy metal capacity of the regional soil in Huzhou to predict the pollution trend [20], and Xu et al. used the comprehensive index method to evaluate the environmental capacity of heavy metals in farmland soils in Fuzhou [21]. However, these studies only explored individual cities of urban agglomerations in the Pearl River Basin and did not systematically explore the soil heavy metal environmental capacity of urban agglomerations in the Pearl River Basin [22,23].

Since 1978, the economy of the Pearl River Basin urban agglomeration has rapidly developed, and its foreign population has also rapidly increased. It is one of the urban agglomerations with the fastest industrialization and urbanization processes in China [24]. The long-term extensive development of labor-intensive industries in this region has aggravated the fragility of the ecological environment, resulting in many “urban diseases” problems, such as soil pollution. Thus, it is becoming more urgent to solve the contradiction between the development and protection of the urban agglomeration in the Pearl River Basin than in other provincial and urban areas. Now, the assessment of the soil heavy metal environmental capacity of the agricultural land in urban agglomerations has become an important and urgent scientific problem. As for the previous studies, they were mainly aimed toward analyzing single provinces or urban areas, and there were only a few works that systematically discussed the soil heavy metal environmental capacity of the agricultural land in urban agglomerations of the Pearl River Basin.

Based on this, this paper systematically discusses the environmental capacity of heavy metals in the agricultural land of the urban agglomeration of the Pearl River Basin by using a GIS spatial analysis, a linear model of the material balance, and the comprehensive index method. The main purposes of the study are as follows. (1) The material balance linear model is used to calculate the soil heavy metal environmental capacity of the agricultural land in the urban agglomeration of the Pearl River Basin. Then, (2) the environmental comprehensive index method was used to evaluate the soil capacity risk and early warning of health risk. Afterward, (3) management countermeasures of the heavy metal pollution in the agricultural land of the urban agglomeration in the Pearl River Basin were put forward. In addition, overall, a guidance and a reference were provided in this study for the health risk warning and treatment of the soil heavy metal environmental capacity problem of the agricultural land in urban agglomerations of the Pearl River Basin.

## 2. Materials and Methods

### 2.1. Study Area

**Figure 1:**
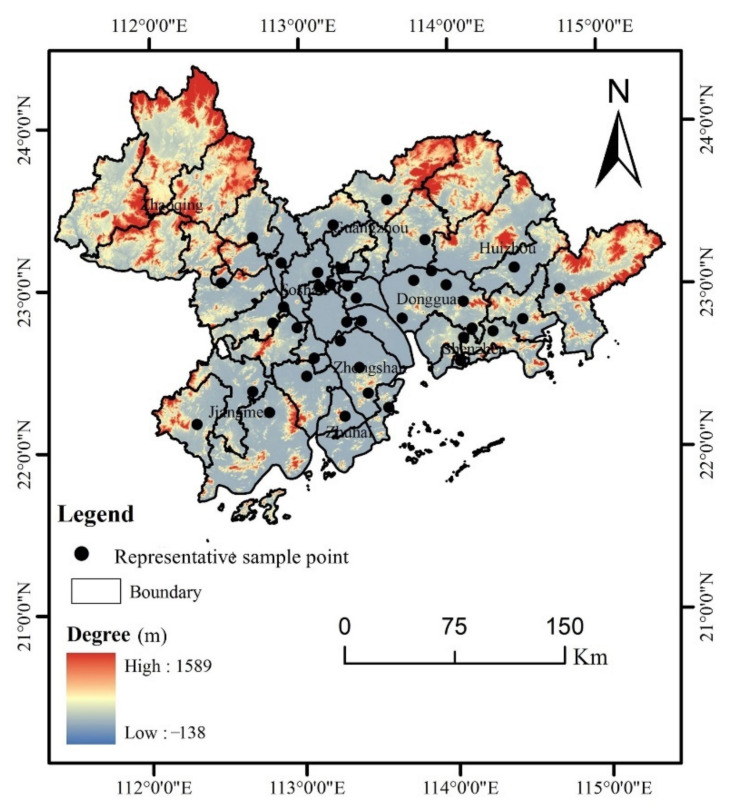
Sampling map of the urban agglomeration in the Pearl River Basin.

The Pearl River Basin urban agglomeration is located in the south–central part of the Guangdong Province and the lower reaches of the Pearl River. It is mainly composed of nine prefecture-level cities: Dongguan, Foshan, Guangzhou, Huizhou, Jiangmen, Shenzhen, Zhaoqing, Zhongshan, and Zhuhai City. Its geographical location is 111°59′42″ E–115°25′18″ E, 21°17′36″ N–23°55′54″ N (Figure 1), and the territory is surrounded by hills and mountains with complex landforms. The total area is about 56,000 square kilometers, and the total population has reached about 70 million. It has a typical subtropical monsoon climate with the same season of rain and heat, with an annual average temperature of 21.9 °C and an annual rainfall of about 1950 mm. Soil formation and distribution are influenced by local soil parent materials, geomorphology and human activities, showing a zonal distribution law, and the main soil types are red soil, lateritic red soil, and latosol [25]. The study area is an important crop planting and aquaculture area in China, and with dense industrial distribution and developed manufacturing industry, which has become one of the largest electronic-led manufacturing centers in the world [26].

### 2.2. Sample Collection and Analysis

According to the “Technical Specifications for Soil Environmental Monitoring (HJ/T 166) [27]”, monitoring points were arranged, and samples were collected. The soil samples were collected from the agricultural land in the Pearl River Basin, which included Dongguan, Foshan, Guangzhou, Huizhou, Jiangmen, Shenzhen, Zhaoqing, Zhongshan, and Zhuhai City, using the grid and random sampling methods. The surface floating soil was removed before sampling, mainly to remove weeds, grass roots, fertilizer lumps and other debris. Additionally, the soil samples (0–20 cm) were then collected in the Pearl River Basin urban agglomeration, avoiding obvious point-shaped pollution areas and newly disturbed soil. The method of multi-point sampling and mixing into a representative sample was adopted, and the final sample of each sample is 1 kg. The representative sample collection points in the study area are shown in Figure 1. In the sampling stage, the reasonable location of the sampling point was determined according to the actual terrain, and the geographical coordinates of the sampling point were determined and recorded by GPS. After natural air drying, the samples were passed through a 100-mesh (<0.84 mm) nylon sieve, and soil intrusions were removed. Anti-pollution measures were taken into consideration during the sample collection, transportation, and processing.

The sample determination was entrusted to Institute of Geophysical and Geochemical Exploration, Chinese Academy of Geological Sciences, in which the As and Hg elements were tested using atomic fluorescence spectrometry (AFS-933, Jitian Corporation, Shanghai, China). The Pb, Zn, Cr, Cu and Ni elements were determined using X-ray fluorescence spectrometry. Additionally, Cd element was determined using electron coupled plasma mass spectrometry (ICP-MS, Agilent 7700X, Agilent Technologies, Santa Clara, CA, USA). Three blank samples and parallel samples were prepared for each batch of soil samples, and the average value was taken as the final content of the sample. In the testing process, the national standard soil reference material (GSS-12) was added for quality control, and the recovery rate of each heavy metal was within the allowable range of the national standard reference material. This study adopted the Chinese local standard (background value of the heavy metals in the soil in the Pearl River Basin (DB44/T1415/2014)) as the background value [28], and it refers to the standard value of the pH < 6.5 in the other categories of the risk screening values in the soil pollution risk Control Standard for Agricultural Land (GB15618-2018) [29] and (GB15618-1995) [30] as the risk reference value, as shown in Table 1.

### 2.3. Methods

#### 2.3.1. Spatial Analysis Method

Inverse distance weight (IDW) [31] means that the reciprocal distance multiplied by the grid method is an increased average interpolation method, which can be transformed or interpolated in a smooth manner (ArcGIS 10.2, accessed on 16 January 2022, http://www.esri.com/software/arcgis, ESRI, Redlands, California, CA, USA). The order parameter controls how the weight coefficient is assigned to a joint order by leaving a grid. The closer data point is given a higher weight distribution. For a smaller order, the weight is more evenly distributed to each data point. The prediction of the soil heavy metal environmental capacity, the single factor, and the comprehensive early warning evaluation of soil heavy metals adopted the interpolation method of a linear, unbiased, and optimal estimation without data transformation, and the original data of the regional variables were used:

(1) $$Z^{*}{(x}_{0})=\sum_{i=1}^{n}\varphi_{i}{\;\times \;Z(x}_{i})$$

where $Z^{*}{(x}_{0})$ is the linear prediction value, ${Z(x}_{i})$ is the observed value, n is the total number of measured samples, i is the samples identifier, and${\;\varphi }_{i}\;$is the optimal weight value that aids in the unbiased prediction with minimum variance.

#### 2.3.2. Linear Model of Material Balance

(1) Static environmental capacity

The static capacity of the soil was determined using the background value and the critical content of the soil environment [18,19]. The larger the difference between the two values, the higher the soil environmental capacity. Since the model parameters could easily be obtained, the study of the soil environmental capacity was often selected:

(2) $${\;Q}_{s}=M\times \left(C_{\mathrm{ic}}{\;-\;C}_{\mathrm{ib}}\right)\;\times \;10^{-6}$$

where Q_s_ is the static soil capacity (kg/hm^2^), M is the soil weight per hectare of the plough layer (2.25 × 10^6^ kg/hm^2^), C_ic_ is the risk reference value (mg/kg), and C_ib_ is the soil background value of the pollutants (mg/kg).

(2) Remaining environmental capacity

The soil residual capacity refers to the difference between the maximum soil load and the pollution status value, and the soil residual capacity was calculated using the following formula [18,19,32]:

(3) $$Q_{i}{=M\;\times \;(C}_{\mathrm{ic}}{-C}_{\mathrm{io}}{)\times 10}^{-6}$$

where Q_i_ is the soil residual capacity (kg/hm^2^), which is the existing environmental capacity of element i in the soil, and C_io_ is the measured value of the element status in the soil (mg/kg).

(3) Dynamic environmental capacity

Soil elements are actual in a dynamic equilibrium process. First, the soil itself contains a certain value, which is the soil background value [17,33]. This value is naturally formed in the soil formation process; although it is in the artificial element cycle, it has relative stability characteristics. Second, the input is a multi-channel and continuous process. Third, the input elements are lost due to underground leakage, surface runoff, and crop absorption. On one hand, these outputs affect the soil elements existence. On the other hand, this existence also affects future input. Since the elements in the soil are in this dynamic equilibrium state, the amount that the soil can hold relative to the soil environmental quality standard is a variable value, as the soil has a variable capacity. According to the material balance linear model, it is assumed that there is a linear relationship between the output of soil pollutants and the content of soil pollutants, and the following formula of the average dynamic annual capacity is obtained using the recursive method year by year [17,32]:

(4) $$Q_{n}=\left[W_{n}{\;-\;W}_{o}{\;\times \;K}^{n}\right]\;\times \frac{1\;-\;K}{{K\;\times \;(1\;-\;K}^{n})}\;{=M\times \;(C}_{\mathrm{ic}}{\;-\;C}_{\mathrm{ib}}{-\;C}_{\mathrm{io}\;}{\times \;K}^{n})\;\times \;\frac{1\;-\;K}{{K\;\times \;(1\;-\;K}^{n})}{\;\times \;10}^{-6}\;$$

where W_n_ is the total amount of heavy metal elements in the soil tillage layer of a region, and a few years later it is expected to be the total amount of heavy metal elements (kg/hm^2^). Wo is the total amount of this element in the tillage layer at the beginning of observation (kg/hm^2^). K is the residual rate, which means that after one year of cultivation, the content of an element in the soil is the sum of the soil content in the previous year and the input in the same year. The migration of heavy metals in soil mainly includes plant absorption, surface runoff and underground leakage, etc. According to studies, the migration of heavy metals in soil is relatively difficult, and the residual rate is generally about 90% [18,19,20]. Due to the lack of experimental data on the soil K value in the Pearl River Basin, it is assumed that the residual rate of various elements in the soil is K = 0.9. Q_n_ is the average dynamic annual capacity kg/(hm^2^a), and n is the number of years of control. Since the set time limit is too short and the change is vague, the follow-up comparative analysis is not operable. However, if the time limit is too long, there would be a big deviation between the predicted results and the real values. It was proposed that the soil dynamic capacity of the cities in the Pearl River Basin urban agglomeration be set for 14 and 24 years, that is, until 2028 and 2038, respectively.

#### 2.3.3. Environmental Capacity Assessment Model

The soil residual environmental capacity of various elements in the Pearl River Basin urban agglomeration was normalized using the range method [17,19,33,34]. In order to highlight the role of the low environmental capacity in the environmental capacity, the improved Nemero comprehensive pollution index was adopted. To illustrate the minimum environmental capacity, the ion was used to replace the original maximum capacity, and the calculation formula is as follows:

(5) $$P=\sqrt{\sqrt{\frac{P_{\mathrm{ave}}^{2}{+P}_{\min}^{2}}{2}}}$$

where P_ave_ is the average value of the normalized environmental capacity of various ions in the i region, P_min_ is the minimum value of the normalized environmental capacity of various ions in the i district, and P is the comprehensive evaluation index of the i district. The comprehensive evaluation and classification standard of heavy metals in the agricultural land in the Pearl River Basin urban agglomeration is shown in Table 2.

## 3. Results and Discussion

### 3.1. Soil Heavy Metal Environmental Capacity

#### 3.1.1. Current Situation of the Environmental Capacity

The static capacities of eight kinds of soil heavy metals in the Pearl River Basin are calculated as shown in Table 3. The overall order of the static capacity of the heavy metals in the soil of the study areas in descending order is Pb, Zn, Cr, As, Hg, Cu, Ni, and Cd, and the average values are 476.25, 302, 235.25, 68.12, 67.29, 63.78, 56.58, 0.37 kg/hm^2^, respectively. As far as the region is concerned, there are some differences in the environmental capacity of soil heavy metals in different cities. The static capacity of heavy metals in Zhongshan City, Foshan City, and Zhuhai City is relatively small, which makes it easy for them to pollute soil. Therefore, soil prevention and control should be strengthened and special actions for soil environmental protection should be carried out in those areas.

Different from the basin area, the static capacity of Pb in the study area is relatively high, while the environmental capacity of Cr, Ni and Cd is much lower than that of Turpan basin [32], which may be related to the metal content of their soil parent materials. Pan et al. believes that this type of industry is also an important reason for the difference in capacity. Their research shows that the industrial pollution in Zhongshan City is mainly concentrated in the northern plain, where there is more electronic processing, toy manufacturing and hardware industries. This is an important reason for its small environmental capacity [33]. The difference between heavy metal environmental capacities in different cities in the study area is mainly because these cities are largely labor-intensive and primary-technology-intensive industries, with more clothing factories, electronics factories and chemical plants.

#### 3.1.2. Spatial Distribution of the Residual Capacity

**Figure 2:**
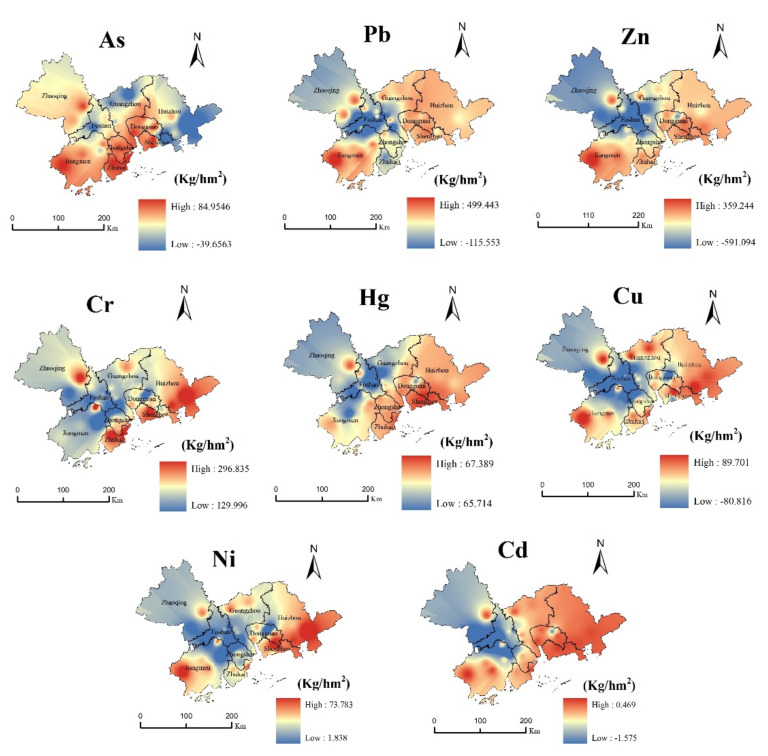
Residual environmental capacity of the heavy metals in the soil of the Pearl River Basin.

According to Formulas (1) and (3), IDW [30] was interpolated to obtain the distribution map of the remaining capacity, as shown in Figure 2. The high-value areas of the soil environmental capacity in the Pearl River Basin Economic Zone were mainly distributed in Jiangmen Enping City, Zhaoqing City, the south of Zhongshan, the north of Shenzhen, and the south of Dongguan, while the low-value areas were mainly distributed in Foshan, Guangzhou, the southeast of Shenzhen, and the south of Huizhou, which are threatened by serious As pollution. The average residual capacity of As in Huizhou is only 28.80 kg/hm^2^, while that in Shenzhen is 37.70 kg/hm^2^. The high-value areas of the Pb capacity were mainly distributed in the north of Guangzhou, Dongguan, Shenzhen, and in the west of Huizhou, while the low-value areas were mainly distributed in the south of Foshan. The average remaining capacity of Foshan was 334.62 kg/hm^2^. The Zn capacity was generally high, and the distribution area was more extensive, where its low-value-capacity areas were mainly distributed in the north of Zhongshan, whose average remaining capacity was 194.03 kg/hm^2^. The high-value areas of the Cr capacity were mainly distributed in the central and eastern parts of Zhaoqing and Zhuhai and in the southeast of Huizhou, while the low-value areas were mainly distributed in the southwest of Foshan, Zhongshan, Shunde District, and Xinhui District, and the soil quality there was poor. The high-value areas of the Hg capacity were mainly distributed in the northeast of Zhaoqing, Shenzhen-Huizhou, and Zhuhai, while the low-value areas were mainly distributed at the junction of Guangzhou and Foshan. The Cu capacity was generally high, the distribution area was scattered, and the low-value areas were mainly distributed in Zhongshan City and in the southwest of Guangzhou. The high-value areas of the Ni capacity were mainly distributed in the southwest of Jiangmen City, Zhaoqing, and Guangzhou, while the low-value areas were mainly distributed in Zhongshan City. The low-value areas of the Cd capacity were mainly distributed in Guangzhou, Zhaoqing, and Zhongshan, whose average residual capacity was 0.13, 0.05 and 0.03 kg/hm^2^, respectively. They showed a gradually increasing outward trend in the form of radiation, even showing a negative value.

We compared the soil environmental capacity of the heavy metals in the Pearl River Basin urban agglomeration with some previous studies, and the results are shown in Table 4. The As of cities in the Pearl River Delta was higher than that of Huzhou in the Yangtze River Delta, and the average As residual capacities of Foshan, Huizhou and Shenzhen was 41.66, 28.80 and 37.70 kg/hm^2^ respectively, lower than that of Nanjing in the Yangtze River Delta. The average residual environmental capacity of Pb and Cr is much lower than that of Nanjing, but higher than that of Huzhou [20]. This also shows that Jiangsu Province is committed to improving the soil ecological environment, which effectively expands the environmental capacity of soil heavy metals Pb and Cr. The environmental capacity of Zn is lower than that of Nanjing and Huzhou, mainly due to the development of hardware industry in the Pearl River Delta region, which accelerates the pollution of Zn to a certain extent and reduces its environmental capacity. However, the residual average environmental capacity of mercury is higher than that of Nanjing and Huzhou. The average residual capacity of Cu in Pingzhou town of Foshan is even negative, so it is necessary to strengthen the prevention and control of Cu pollution. The average residual environmental capacity of Ni is higher than that of Nanjing [19]. The Cd residual environmental capacity of cities in the Pearl River Basin is lower than that in Nanjing and Huzhou in the Yangtze River Basin [19,20]. It indicated that these elements, which accumulated with economic development, may be related to the rapid urbanization and exponential industrial agglomeration in these areas after the Chinese reform and opening-up [35,36].

#### 3.1.3. Prediction of the Environmental Capacity

**Figure 3:**
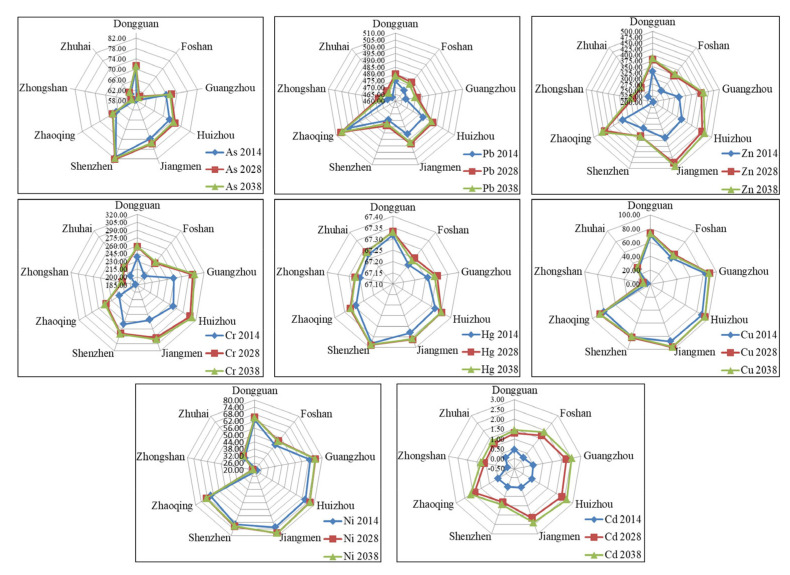
Dynamic environmental capacity in the soil in the Pearl River Basin.

The static and residual capacities of soil do not take into account the output and self-purification, so it is incomplete to only consider the static capacity in soil environmental capacity studies. Therefore, based on Formulas (1) and (4), the environmental capacity of 8 kinds of heavy metals in the Pearl River Basin urban agglomeration was predicted, respectively, and the predicted results are shown in Figure 3. The results of dynamic environmental capacity showed that the environmental capacity of Zn, Cr and Cd in the study area greatly increased. For 2038, predictions showed that the average capacity of Zn, Cr and Cd in the study area increased by 88.90, 31.43 and 1.53 kg/hm^2^, respectively. The growth rate was relatively fast from 2014 to 2028 and slowed down gradually from 2028 to 2038, which may be related to the industrial upgrading and industrial structure reform of Guangdong Province in recent years. The dynamic environmental capacity of different elements greatly varies, and so does the rate of consumption, where the environmental capacity of the heavy metals in the soil of the urban agglomeration in the Pearl River Basin showed an overall upward trend after 14 and 24 years, and some urban elements showed fluctuations. For example, the environmental capacity of the soil Cu and Ni in Shenzhen in 2038 was predicted to be slightly lower than that in 2028, indicating that the soil self-purification capacity of the Cu and Ni heavy metals in this area was limited. There is an urgent need to strengthen the prevention and control of pollution. The growth rate of the environmental capacity of heavy metals in the soil from 2014 to 2028 was higher than that from 2028 to 2038, and the environmental capacities of the Zn and Cu showed the clearest values. From 2014 to 2038, the environmental capacity of the soil Cd in the study area was lowest, where it was less than 3.0 kg/m^2^, and it also exceeded the standard of Cd pollution.

These findings are different from Ma’s research results [17], which show that, under the condition of maintaining the current value of other heavy metal elements in the soil of Jinghe County of Xinjiang without pollution, with the effect of soil self-purification, the current situation of environmental pollution will tend to improve. However, we believe that pollution control measures should be taken to alleviate the heavy metal pollution in the study area, so as to expand the heavy metal mitigation capacity. The study of Pan et al. [33] agrees that the capacity of Zn and Cu in soil heavy metals in Zhongshan has a strong potential for growth. Meanwhile, Zhongshan City has also taken a series of measures to alleviate and prevent heavy metal pollution. For example, Zhongshan City has issued Zhongshan City’s Advantage Traditional Industries Transformation and Upgrade Action Plan (2018–2022). Emphasis is placed on promoting the transformation and upgrading of traditional industries, such as home appliances, hardware, lighting and furniture, and promoting enterprises to carry out digital, networked, intelligent and green technological transformations, speed up the optimization and upgrading of superior traditional industries, and cultivate a new momentum for development [37]. Therefore, we believe that the Pearl River Delta region should continue to strengthen industrial upgrading, strengthen the development of low-carbon industries, and solve the contradiction between economic development and environmental protection.

### 3.2. Evaluation and Health Risk Warning of the Heavy Metal Environmental Capacity

**Figure 4:**
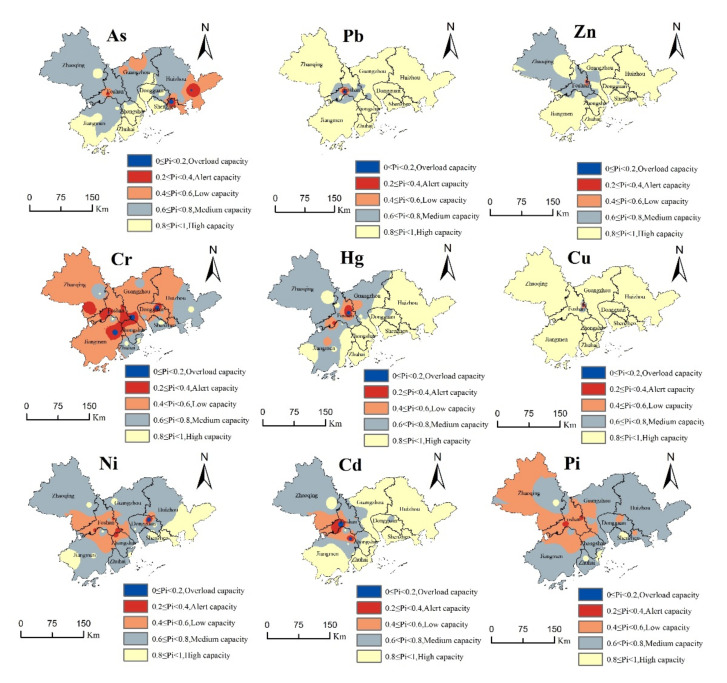
Evaluation map of the soil heavy metal environmental capacity.

In this study, based on Formulas (1) and (5), the visual early warning of single factors and the comprehensive environmental capacity risks of eight kinds of heavy metals in the study area were carried out, as shown in Figure 4. The comprehensive soil quality index of the Pearl River Basin was 0.64; the pollution level was grade IV, which belongs to the medium capacity; and the corresponding health risk was a mild risk. Combined with Figure 3 and Figure 4, the dynamic capacities of the soil heavy metals in each city showed an upward trend from 2014 to 2038, but they did not exceed the soil environmental quality standards, and the regional soil environmental quality was better. According to Figure 4, where it can be seen that there are some differences in the different heavy metal elements in different cities. The average Pi values of heavy metal As in Dongguan City and Zhongshan City are 0.88 and 0.85. The capacity level is high and the health risk is risk-free. The capacity level of As in Huizhou is relatively low, with an average of 0.55 and a moderate health risk. The environment of Pb in the study area is higher, but there are also low values, such as Gaoming District of Foshan City. At the same time, the environmental capacity of Hg and Cd is also relatively low, with an average Pi value of 0.5, which bears moderate health risks. This may be related to its early rapid development, but in recent years, Gaoming District pays attention to ecological restoration and has become one of the top 100 areas of scientific and technological innovation. The Cr capacity of each city in the study area is relatively low, which may be related to the rapid development of hardware and chemical industry in the Pearl River Delta. Except Shenzhen, Dongguan and Huizhou, other cities have low a Ni environmental capacity and certain health risks. Except Foshan, the average Pi value of Zn in all cities was higher than 0.8, indicating a high capacity and no health risk.

Generally, the soil environmental capacities of all the kinds of heavy metals in Zhaoqing, Huizhou, Jiangmen, and Zhuhai were mostly surplus, while the soil environmental capacity of the Guangzhou–Foshan belt showed a serious overload phenomenon. The environmental capacities of As, Cr, and Cd in the cities were low, showing greater health risks than other heavy metal elements, while the health risk prevention measures should be considered in some areas, such as the Nansha District of the Guangzhou and Gaoming District of Foshan. The heavy metal soil pollution in the Pearl River Basin region showed a law of regional difference, and the heavy metal soil pollution in the Xijiang River Basin was much higher than that in the Dongjiang River Basin, whose health risk was also higher than that of Dongjiang. Since the population of the main urban area was more concentrated, and the municipal solid waste was much higher in the main area than in the surrounding areas, the soil environmental capacity in the main urban area was lower than that in the surrounding areas. With the high level of industrialization in the region and the rapid development of industrialization, the Cd, Cu, and Ni elements rapidly accumulated in the soil. Pan et al. [33]. believe that this is mainly related to the combustion of fossil fuels in industrial production and the emission of the industrial “three wastes”. With the continuous acceleration of urbanization, land use has been drastically changed and transferred, and the safety utilization rate of the contaminated land is low.

We discussed the environmental capacity risk level of the study area with reference to previous studies , as shown in Table 5. The Pi values of Dongguan, Foshan, Guangzhou, Huizhou, Jiangmen, Shenzhen, Zhaoqing, Zhongshan and Zhuhai were 0.76, 0.44, 0.60, 0.71 and 0.73, respectively. The environmental capacity of heavy metals in Zhuhai was the highest, but the environmental capacity level of Foshan was the lowest, which required a capacity early warning, and the health risk was a moderate risk. Additionally, other cities in the study area had a mild early warning. This was mainly because the industry of Zhuhai is mainly concentrated in Jinwan District and Doumen District, with more electronic processing and clothing processing, less hardware and chemical industries, and Zhuhai is committed to developing seaside tourism and paying attention to environmental protection, so that it has more environmental capacity and less risk of human health. However, in the early stage of reform and opening up, Foshan City was rich in mineral resources and introduced a large number of hardware manufacturing and chemical industries, resulting in relatively serious pollution and a low environmental capacity. However, in the later period, the Government realized the importance of protecting the ecological environment and implemented a series of measures to reduce water, soil and gas pollution to qualitatively improve the ecological environment. According to Table 5, the average capacity grade of the surface soil in Huzhou City in the Yangtze River Basin urban agglomeration is in the medium-capacity area, and most of the soil is in the medium- and high-capacity areas. Chen et al. believe that the main source of heavy metals in the topsoil of Huzhou is the discharge of three wastes from the electroplating and battery enterprises in the area [20]. Additionally, the environmental capacities of eight kinds of heavy metals in the Lishui District of Nanjing were generally at the medium-capacity level, with a comprehensive index of 0.9, which showed that the environmental capacity there was sufficient [19]. From the spatial distribution perspective, the medium-capacity area was large, while the high-capacity, low-capacity, warning, and overloaded areas were small. There were capacity overloads for As, Cu, and Ni, and there were no low-capacity, warning, and overloaded areas for Cr. Additionally, there were no warning and overloaded areas for Pb. Fu et al. believe that there are many kinds of heavy metal pollution in some areas of the Lishui District of Nanjing City, such as the As, Cd, and Cu pollution in the south of the Yaojia Reservoir and the As, Cd, and Ni pollution in the north of the Weishan Reservoir, which might be related to their pollution sources, such as the local chemical production and mineral exploitation industries, which may lead to a variety of heavy metals [19]. The environmental capacity of the study area is relatively low, which is mainly due to the industrial characteristics of the city, such as the development of traditional light industry in the Pearl River Delta, and the pollution mainly comes from industrial waste and human activities [33].

### 3.3. Suggestions on the Implementation of the “Soil Safety”

**Figure 5:**
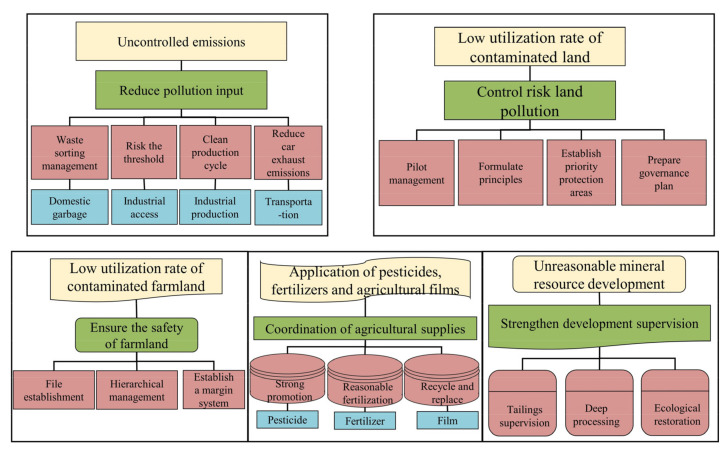
The main contents of the countermeasures of the “soil safety” of the Pearl River Basin.

With reference to the evaluation index system and the implementation plan for the construction of Beautiful China, which were issued by the National Development and Reform Commission of China in 2020 [38]. The evaluation index system for the construction of Beautiful China, which is composed of fresh air, clean water, soil security, good ecology, and clean human settlements, in addition to 22 specific indicators based on the “soil security” index, the idea for the treatment of the soil heavy metal pollution in agricultural land in the urban agglomeration of the Pearl River Basin was put forward (Figure 5).

#### 3.3.1. Reducing the Input of the Polluting Raw Materials

The main results are as follows: (1) For solid wastes, such as domestic waste, we should strengthen the advertising of garbage classification, promote the recycling of domestic waste, and promote the resourceful and harmless treatment of municipal solid waste. Additionally, some disposal methods, such as on-site stacking and simple landfills, should be prohibited to avoid the soil pollution caused by garbage disposals [39]. (2) For industrial access, entry thresholds for enterprises with polluting production capacities should be raised to prevent the new industrial projects from causing new soil pollution. Additionally, the supervision and assessment of the elimination of poor production capacity should be strengthened, and the blacklisted enterprises that have been eliminated within a time limit should be announced on a regular basis. In addition, “equal replacement” or “reduction replacement” should be implemented for new industries that emit heavy metals. (3) For industrial production, the supervision of the key polluting industries should be strengthened, and the pollution control of the heavily polluting industries, such as plastics and electroplating, in Zhongshan and other cities, should be strengthened. Moreover, circular economy and cleaner production should be carried out. (4) For transportation, the vehicle exhaust emissions should be controlled, and relevant management policies should be formulated, such as restricting the number and frequency of trips of non-local license plates in other cities in the Pearl River Basin, asking vehicle owners to install exhaust purifiers, and encouraging the purchase of electric vehicles, so as to reduce the concentration of heavy metals in the atmosphere [40].

#### 3.3.2. Managing and Controlling the Risks of Contaminated Lands

(1) Pilot management of the environmental supervision of the contaminated plots should be carried out in Guangzhou, Shenzhen, Foshan, and Zhongshan. All localities should take the original sites of the chemical, metal smelting, pesticide, electroplating, and dangerous chemical enterprises that have been closed, bankrupt, and relocated as objects to organize and carry out soil environmental investigation and risk assessment and to establish the treatment and restoration of the contaminated sites. (2) The “who pollutes, who treats” principle should be followed, and the units that cause site pollution should bear the responsibilities of soil environmental investigations, risk assessments, treatments, and restorations [41]. (3) An environmental management system of the priority areas should be established for soil environmental protection, strengthening the investigation, controlling the pollution sources in priority areas, and carrying out a pilot demonstration of the soil environmental protection and comprehensive treatment in Dongguan City, which is necessary to investigate the production and discharge of pollutants and the implementation of daily regulatory measures [42]. (4) The pollution source control plan and the demonstration project of the treatment should be carried out.

#### 3.3.3. Ensuring the Safe Management of Cultivated Lands

(1) Archives and cards should be established, each county should be taken as a unit, the investigation and monitoring of the soil heavy metal pollution in agricultural producing areas should be carried out, and soil environmental quality files in agricultural areas should be established. (2) The grading management system of the cultivated land soil pollution should be carried out, and the cultivated land should be further divided into suitable areas, monitoring areas, and prohibited production areas. For the uncontaminated cultivated soil, a permanent protection system should be established, and effective measures, such as returning straw to the field, should be applied to increase the soil organic matter. For lands with a low degree of pollution, which can still be used as cultivated land, certain measures, such as planting structure adjustment, agronomic regulation, soil pollution control, and remediation, should be applied to ensure the safe use of the cultivated land. Additionally, rotation and fallow measures should be taken for the cultivated land that is seriously polluted and difficult to repair. For the land that is not suitable for agriculture, land use should be adjusted according to the law, and the prohibited production areas of agricultural products should be divided [43]. (3) In accordance with the “concentration into a piece, dynamic adjustment, and no reduction in the total amount” principle, a “deposit” system for the treatment of heavy metal pollution in the cultivated soil was formulated, and the county government set up a fund management committee. Enterprises that shall cause pollution in the surrounding areas should pay a deposit for environmental pollution according to the pollution situation, which can be used to control and repair the cultivated soil [44].

#### 3.3.4. To Co-Ordinate the Use of Agricultural Materials

(1) The utilization rate of the pesticides should be improved; the application and promotion of high-efficiency, low-toxicity, and biological pesticides in the rural areas of the three major urban agglomerations should be carried out; and green prevention, control technology, and professional unified control should be vigorously promoted. Biological genetic engineering and natural enemies for control diseases, such as transgenic technology, should be used, and vaccines or antibodies should be added to crops to partially replace the use of pesticides. The investment in science and technology should be increased, and the industrial production of organic pesticides should be promoted [45]. (2) Chemical fertilizers should be scientifically and rationally applied, the utilization rate of chemical fertilizers should be improved, and the application of fertilizers with excessive heavy metals should be prohibited. The use of microbial fertilizers, livestock, and poultry manure should be harmless, and such fertilizers should be tested before application so as to meet the necessary standards. To develop new types of organic fertilizers, the waste of food processing plants should be used as a raw material for production of organic fertilizers, which are rich in nitrogen, phosphorus, potassium, and trace elements, as well as animal protein and fat residues. (3) The recovery rate of agricultural films should be enhanced, the recovery and comprehensive utilization of waste agricultural films should be encouraged, and a recycling system for waste, such as pesticide packaging containers and agricultural films, should be established. Additionally, the use of disposable foamed plastic tableware and ultra-thin plastic bags should be prohibited, pilot projects for the classified recovery of waste plastics should be carried out, and agricultural waste should be prevented from polluting the soil. In addition, degradable materials should be used instead of plastic films, such as plant fiber powders and amine hot pressing technology; one-year-old plant fiber powder and special additives should be used; and the production process should create less environmental pollution [44].

#### 3.3.5. Strengthening the Supervision of Mineral Development

(1) An eco-environmental supervision and daily inspection system should be strictly implemented, the safety supervision of the tailing reservoirs should be strengthened, and the soil pollution caused by safety accidents should be prevented. Additionally, illegal activities in the development and utilization of the mineral resources, such as indigenous metallurgy and mercury smelting, should be strictly prevented, and regional listing supervision should be carried out. (2) The heavy metal “three wastes” pollutants in the mining area should be properly dealt with and the enterprises should be urged to implement measures for reducing and controlling the discharge of heavy metals and persistent organic pollutants. Additionally, advanced treatments should be encouraged on the basis of stable discharge standards. (3) The environmental restoration of the mining areas should be paid attention to, the surface layer of the soil should be removed before mining, and then the abandoned soil layers should be covered [45]. In addition, comprehensive work concerning the microbial improvement should be carried out, the soil structure should be optimized, and the re-breeding of the vegetation in mining areas should be ensured.

## 4. Conclusions

In conclusion, based on a GIS spatial analysis, the material balance linear model, and the comprehensive index method, this study systematically discussed the environmental capacity of heavy metals in the agricultural lands in the urban agglomeration of the Pearl River Basin. We concluded that the static capacity of heavy metals in Foshan City, Zhongshan City, and Zhuhai City, from the Pearl River Basin urban agglomeration, is smaller than that in other cities. Additionally, the dynamic environmental capacity of each city in the study area showed an overall upward trend, and some urban elements showed fluctuations. The best remaining capacity was that of Pb and Cu, while the capacities of As, Zn, Hg, and Cd were relatively poor in the Cr and Ni study area. The comprehensive soil quality index of the Pearl River Basin was 0.64, and the pollution level was grade IV, which belongs to the medium capacity, but there are still some health risks that showed clear regional differences. This paper preliminarily puts forward a train-of-thought framework for “soil safety”, which should include at least six aspects: reducing the input of the pollution source materials, controlling the risk of the contaminated land, improving the graded management of the cultivated land, coordinating the use of agricultural materials, and strengthening the supervision of mineral development.

It is worth noting that there are still some limitations to this study. Due to the lack of research on the soil heavy metal environmental capacity of the urban agglomerations in the Pearl River Basin, China. The lack of data concerning the soil environmental capacity of urban agglomerations in the Pearl River Basin, and other urban agglomerations have not yet been considered and need to be further discussed in the future. Overall, soil safety is an important evaluation index for the construction of Beautiful China, and the evaluation and treatment of the heavy metal soil environmental capacity is an urgent scientific problem at present. There is an urgent need to systematically and deeply analyze the complex mechanism of soil pollution in urban agglomerations from a multi-disciplinary perspective, accurately locate sources, and strictly control the discharge of pollutants.

## Figures and Tables

**Table 1:** Background and reference values of heavy metals in soil of Pearl River Basin (mg/kg).

Element	Background Value	Reference Value
As	25.00	40.00
Pb	60.00	250.00
Zn	97.00	200.00
Cr	77.00	150.00
Hg	0.13	30.00
Cu	32.00	50.00
Ni	28.00	40.00
Cd	0.11	0.30

**Table 2:** Comprehensive evaluation of the soil quality in the Pearl River Basin.

Level	Pi	Capacity Level	Health Risk Level
Ⅰ	0 ≤ Pi < 0.2	Overload capacity	Extreme risk
Ⅱ	0.2 ≤ Pi < 0.4	Alert capacity	Severe risk
Ⅲ	0.4 ≤ Pi < 0.6	Low capacity	Moderate risk
Ⅳ	0.6 ≤ Pi < 0.8	Medium capacity	Mild risk
Ⅴ	0.8 ≤ Pi < 1	High capacity	No risk

**Table 3:** Static capacity in the soils of the cities in the Pearl River Basin (kg/hm^2^).

City	As	Pb	Zn	Cr	Hg	Cu	Ni	Cd
Dongguan	70.42	474.75	328.50	238.50	67.32	69.98	62.78	0.46
Foshan	58.05	470.25	261.00	207.00	67.21	49.05	47.92	0.21
Guangzhou	69.75	468.00	319.50	258.75	67.26	84.60	69.30	0.49
Huizhou	72.90	483.75	348.75	267.75	67.32	88.65	71.32	0.55
Jiangmen	73.80	486.00	362.25	256.50	67.33	87.98	72.45	0.52
Shenzhen	81.22	474.75	319.50	265.50	67.38	81.00	69.75	0.49
Zhaoqing	66.82	499.50	353.25	227.25	67.29	79.88	64.58	0.49
Zhongshan	58.50	465.75	195.75	189.00	67.25	4.95	17.78	−0.12
Zhuhai	61.62	463.50	229.50	207.00	67.27	27.90	33.30	0.21

**Table 4:** Residual capacity in the soils of the cities in the Pearl River Basin (kg/hm^2^).

Urban Agglomeration Area	City	As	Pb	Zn	Cr	Hg	Cu	Ni	Cd	References
The Pearl River Basin	Dongguan	69.76	457.48	291.55	232.88	67.24	53.38	59.41	0.38	This study
	Foshan	41.66	334.62	17.65	189.77	66.51	−3.79	35.84	0.56	
	Guangzhou	43.79	413.21	214.85	196.04	66.96	39.32	43.51	0.13	
	Huizhou	28.80	440.33	264.08	252.68	67.17	69.68	65.63	0.38	
	Jiangmen	57.79	449.63	282.49	205.46	67.05	46.58	53.44	0.00	
	Shenzhen	37.70	452.31	280.75	239.91	67.37	58.39	65.76	0.41	
	Zhaoqing	65.14	468.46	212.40	224.11	67.14	44.33	50.29	0.05	
	Zhongshan	65.48	439.05	194.03	216.15	67.19	13.34	42.75	0.03	
	Zhuhai	76.84	400.84	288.12	277.99	67.24	67.39	60.98	0.34	
The Yangtze River Basin	Nanjing	42.55	493.58	317.24	400.57	0.51	52.97	31.08	0.46	[19]
	Huzhou	21.80	151.00	486.00	85.70	1.92	173.00	61.20	1.70	[20]

**Table 5:** The soil environmental capacity level of agricultural land in urban agglomeration area.

Urban Agglomeration Area	City	Pi	Capacity Level	Risk Level
The Pearl River Basin	Dongguan	0.76	Medium capacity	Mild risk
	Foshan	0.44	Low capacity	Moderate risk
	Guangzhou	0.60	Medium capacity	Mild risk
	Huizhou	0.71	Medium capacity	Mild risk
	Jiangmen	0.63	Medium capacity	Mild risk
	Shenzhen	0.74	Medium capacity	Mild risk
	Zhaoqing	0.70	Medium capacity	Mild risk
	Zhongshan	0.67	Medium capacity	Mild risk
	Zhuhai	0.87	Medium capacity	Mild risk
The Yangtze River Basin	Nanjing	0.91	High Capacity	No risk
	Huzhou	0.88	High Capacity	No risk

## Data Availability

The data that support the findings of this study are available from the corresponding author, M.Z. (zengmin1982@sina.com), upon reasonable request.
